# A Case of Trigeminal Neuralgia in an Adult Patient With Lambdoid Synostosis

**DOI:** 10.7759/cureus.56918

**Published:** 2024-03-25

**Authors:** Shunsuke Izumi, Tatsya Takezaki, Yuki Takeshima, Tadashi Hamasaki, Akitake Mukasa

**Affiliations:** 1 Neurosurgery, Kumamoto University Hospital, Kumamoto, JPN

**Keywords:** craniofacial deformity, trigeminal nerve decompression, cranial neurosurgery, neurosurgery, neurovascular compression, unilateral lambdoid synostosis, trigeminal neuralgia

## Abstract

Trigeminal neuralgia (TN) is characterized by sudden, brief intense pain in the distribution of the unilateral trigeminal nerve (TGN). Neurovascular compression (NVC) of the TGN is the most common cause of TN. Recent studies have suggested that a structural anomaly of the posterior cranial fossa might be involved in the development of TN, and several studies have documented the association between NVC-related TN and congenital posterior cranial deformities in adults. We present the case of a 56-year-old woman with NVC-related TN and unilateral lambdoid synostosis (ULS), along with a literature review, to investigate the relationship between TN and structural anomalies of the posterior fossa. This is the first report of TN in an adult with ULS. Mild and asymptomatic cases of lambdoid synostosis might have a higher incidence of NVC-related TN in association with posterior cranial fossa deformities.

## Introduction

Trigeminal neuralgia (TN) is a condition characterized by paroxysmal, short, sharp, intense attacks of pain involving one or more divisions of the unilateral trigeminal nerve (TGN). The most common cause of TN is compression of the fifth cranial nerve at its root entry zone (REZ) by a vascular loop [1]. For the diagnosis of TN, MRI is of great use when it shows neurovascular compression (NVC) of the TGN in addition to basic assessments such as obtaining a medical history and performing a neurological examination. Although the increasing curvature of the small artery caused by age-related atherosclerosis has appeared to be a main culprit of TN in adults, several studies recently suggested that a structural anomaly of the posterior cranial fossa could play a key role in facilitating the development of TN [2-4].

Craniosynostosis is a rare developmental anomaly that occurs because of premature fusion of fibrous cranial sutures, causing an aberrant growth pattern of the young infant skull. Craniosynostosis patients often have a higher risk of impaired cognitive development due to increased intracranial pressure (ICP). Lambdoid synostosis is the rarest of all types of craniosynostosis. In the case of unilateral lambdoid synostosis (ULS), posterior plagiocephaly is produced. Relevant clinical profiles relating to the deformation of the posterior cranial fossa have been described in a few reports [5].

There have been no reports showing an association between TN and craniosynostosis. We report a case of NVC-related TN to examine the possible mechanism of TN with structural anomalies of the posterior fossa along with a review of the literature.

## Case presentation

**Figure 1:**
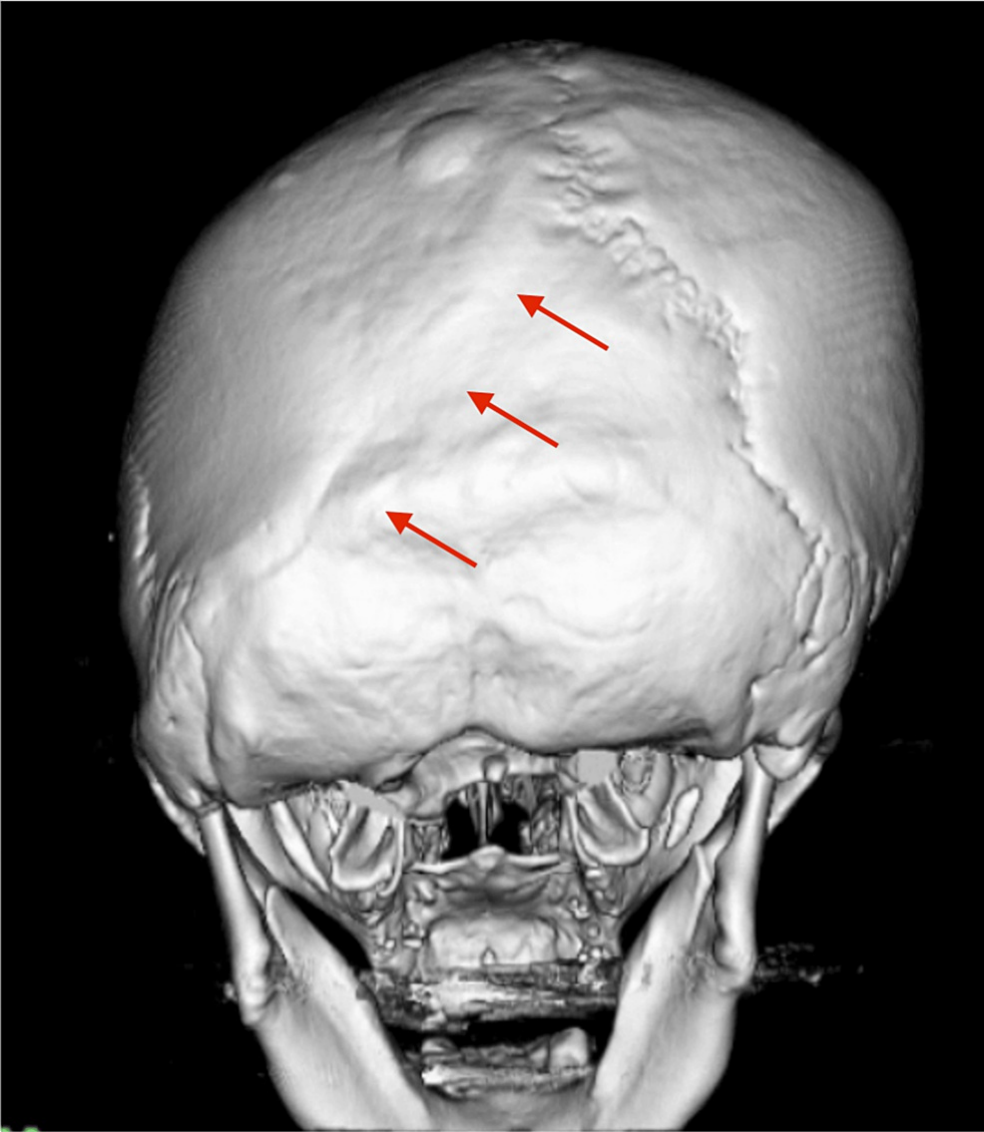
CT three-dimensional reconstruction. The CT scan shows a deformity of the head without clear identification of the left lambdoid suture. CT: computed tomography

**Figure 2:**
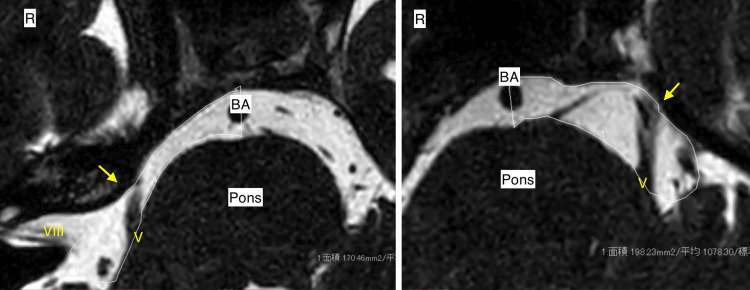
MRI T2-weighted images. MRI scan demonstrates a very narrow space around the right cerebellopontine cistern with a bulky shape of the petrous bone. MRI: magnetic resonance imaging; BA: basilar artery; V: cranial nerve V; VIII: cranial nerve VIII

**Figure 3:**
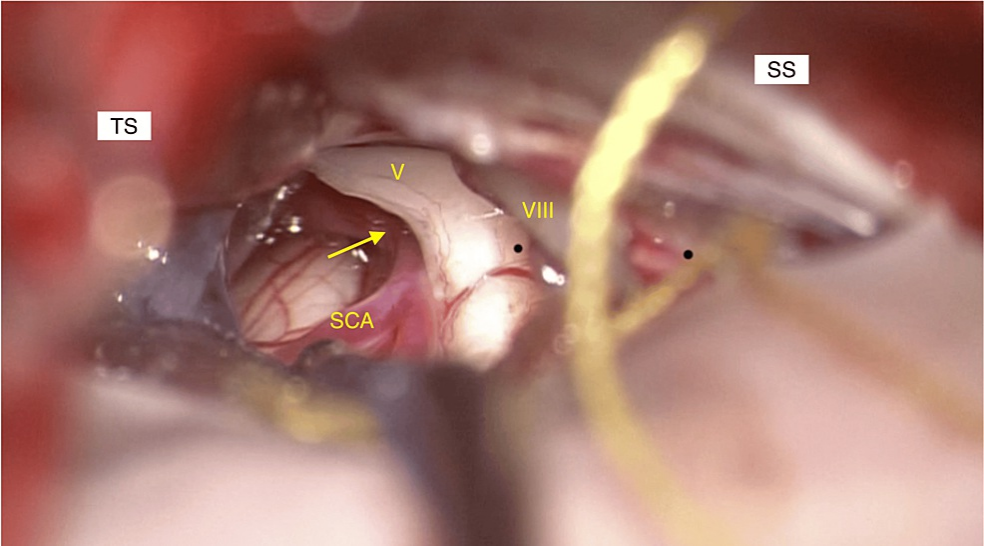
The operative field surrounding the trigeminal nerve. The trigeminal nerve, running from the caudal side to the cranial side, directly compressed by the right SCA. TS: transverse sinus; SS: sigmoid sinus; V: cranial nerve V; VIII: cranial nerve VIII: SCA, superior cerebellar artery

A 56-year-old woman had typical TN affecting the right facial region along the maxillary and mandibular branches of the TGN for almost two years. The neuralgia attacks were triggered by specific movements or tactile stimuli, including eating or toothbrushing. Symptoms improved with an anticonvulsant, carbamazepine, but gradually worsened. One year later, worsening pain caused her to visit a hospital for a detailed examination. Her general physical and neurological condition was good except for the TN. A trigger point for the TN was found in the right maxillary region. CT showed a deformity of the head without clear identification of the left lambdoid suture, indicating ULS as described above (Figure 1). MRI demonstrated that the right TGN was compressed by the right superior cerebellar artery at its REZ, with a quite narrow space around the right cerebellopontine cistern with a bulky shape of the petrous bone, possibly coming from asymmetric structural development resulting from the effect of ULS (Figure 2). She experienced no significant relief after taking 800 mg, divided into two doses per day, of carbamazepine. A standard microvascular decompression (MVD) of the TGN was performed in a right retrosigmoid craniotomy. The fifth cranial nerve was directly compressed by the right superior cerebellar artery (SCA) from the caudal side, and the indentation of the nerve root was clearly visible (Figure 3). The surrounding arachnoid of the right SCA was carefully dissected, and the artery and the fifth cranial nerve were fully detached from each other, even though the following anomalies were observed: (1) the operative field surrounding the fifth cranial nerve was much tighter than expected; (2) the right internal acoustic meatus deviated toward the cranial and ventral sides; and (3) the facial nerve was aberrantly running from the caudal side to the cranial side (Figure 3). The patient’s postoperative course was uneventful without any complications, with no recurrence of TN for at least one year.

## Discussion

The most common cause of TN is compression of the fifth cranial nerve at its REZ by a vascular loop [1]. Although the role of anatomical structures in the pathogenesis of TN has not been well understood, previous studies suggested a relationship between susceptibility to TN and canonical structural anomalies, such as cases of TN associated with posterior cranial deformities [6,7], among which Smartt et al. reported that the petrous ridge angle was larger in the lambdoid synostosis group on the unaffected side [8]. In the present case, the following anatomical parameters were observed: the length of the TGN was shorter on the unaffected side (8.1 mm vs. 9.2 mm); the calculated area of the cistern was narrower (170.2 mm^2^ vs. 198.0 mm^2^) on the unaffected side at the level of the fifth cranial nerve; and the angle between the ventral median line and the petrous ridge (petrous ridge angle) was larger on the unaffected side (131° vs. 122°), although deviation of the median line of the patient’s skull impeded correct assessment of the actual petrous angle.

Craniosynostosis is a developmental anomaly that occurs as a consequence of premature fusion of the cranial sutures, whose underlying mechanism is not well understood. The prevalence of craniosynostosis is quite rare, reported to be 1 in 2,100-2,500 births [9]. Premature closure of the sutures prompts the parts of the skull to grow perpendicular to the unclosed sutures, as the developing brain still grows and expands in the direction of lower resistance. This abnormal growth may alter the shape of the skull to various degrees. In mild cases, most patients can achieve normal brain development despite the aberrant head shape, whereas in more severe cases, increased ICP could disturb normal brain development to cause permanent neurological deficiencies [10]. The location of the affected sutures determines the shape and appearance of the child’s head, which leads to a precise diagnosis of craniosynostosis. Lambdoid synostosis, as a result of the premature closure of the lambdoid suture, is the rarest of all types of craniosynostosis, with a frequency believed to be less than 0.3 in 10,000 births. Clinical features include specific abnormalities such as unilateral occipital flattening, tilting of the head, and inferior deviation of the enlarged ipsilateral ear [5]. Lambdoid synostosis can cause the bony structure of the posterior fossa to drift toward the contralateral side of the fused suture. This deformity may cause a petrous bone asymmetry, where the enlargement of the middle cranial fossa on the contralateral side can create a bulging petrous bone with a widening of the petrous ridge angle [5]. A wider petrous angle can create a tighter cerebellopontine cistern to draw the branches of the upper basilar artery to the vicinity of the REZ of the TGN, which can increase the frequency of TN in patients with lambdoid synostosis. In this case, the right internal acoustic meatus deviated toward the cranial and ventral sides, and the facial nerve was aberrantly running from the caudal side to the cranial side. These unusual operative findings could have caused anatomical disorientation. For the safety of surgical treatment of cases with structural anomalies, it is necessary to prepare both functional monitoring such as auditory brain response, nerve integrity monitor, and neuronavigation system.

Bečulić et al. reported the usefulness of preoperative planning with three-dimensional (3D) printing in surgical treatments for craniofacial reconstruction patients [11]. Advances in 3D imaging and printing can aid in more accurate and safer procedures for patients. Several reports also have documented the benefits of 3D printing for patients undergoing MVD [12,13]. In this case, which includes both of them, we think 3D printing would be of great use both pre- and intraoperatively.

In fact, there are scattered reports of NVC-related TN in adult patients associated with diseases involving posterior cranial deformities [6,7]. Based on these considerations, mild lambdoid synostosis cases, especially adults who were asymptomatic in childhood, may experience more frequent TN due to NVC associated with deformities of the posterior cranial fossa. This is the first report of TN in an adult with ULS.

## Conclusions

This is the first report of TN in an adult with asymptomatic ULS. Lambdoid synostosis is the rarest type of craniosynostosis and involves premature closure of the lambdoid suture, and the bony structure of the posterior cranial fossa might drift toward the contralateral side of the fused suture. This can lead to petrous bone asymmetry in which the middle cranial fossa becomes enlarged on the contralateral side and causes the top of the petrous bone to bulge and the petrous ridge angle to widen. Thus, mild and asymptomatic cases of lambdoid synostosis might have a higher incidence of NVC-related TN in association with posterior cranial fossa deformities. For the safety of surgical treatment of cases with structural anomalies, it is necessary to prepare both functional and anatomical monitoring.

## References

[1] Jannetta PJ, McLaughlin MR, Casey KF (2005). Technique of microvascular decompression. Technical note. Neurosurg Focus.

[2] Brinzeu A, Dumot C, Sindou M (2018). Role of the petrous ridge and angulation of the trigeminal nerve in the pathogenesis of trigeminal neuralgia, with implications for microvascular decompression. Acta Neurochir (Wien).

[3] Horínek D, Brezová V, Nimsky C (2009). The MRI volumetry of the posterior fossa and its substructures in trigeminal neuralgia: a validated study. Acta Neurochir (Wien).

[4] Rasche D, Kress B, Stippich C, Nennig E, Sartor K, Tronnier VM (2006). Volumetric measurement of the pontomesencephalic cistern in patients with trigeminal neuralgia and healthy controls. Neurosurgery.

[5] Matushita H, Alonso N, Cardeal DD, Andrade FG (2014). Major clinical features of synostotic occipital plagiocephaly: mechanisms of cranial deformations. Childs Nerv Syst.

[6] de Almeida Holanda MM, Pereira Neto NG, de Moura Peixoto G, Pinheiro Santos RH (2015). Trigeminal neuralgia secondary to basilar impression: a case report. J Craniovertebr Junction Spine.

[7] Gnanalingham K, Joshi SM, Lopez B, Ellamushi H, Hamlyn P (2005). Trigeminal neuralgia secondary to Chiari's malformation--treatment with ventriculoperitoneal shunt. Surg Neurol.

[8] Smartt JM Jr, Elliott RM, Reid RR, Bartlett SP (2011). Analysis of differences in the cranial base and facial skeleton of patients with lambdoid synostosis and deformational plagiocephaly. Plast Reconstr Surg.

[9] Boulet SL, Rasmussen SA, Honein MA (2008). A population-based study of craniosynostosis in metropolitan Atlanta, 1989-2003. Am J Med Genet A.

[10] Kajdic N, Spazzapan P, Velnar T (2018). Craniosynostosis - recognition, clinical characteristics, and treatment. Bosn J Basic Med Sci.

[11] Bečulić H, Spahić D, Begagić E (2023). Breaking barriers in cranioplasty: 3D printing in low and middle-income settings-insights from Zenica, Bosnia and Herzegovina. Medicina (Kaunas).

[12] Colaguori F, Marin-Mera M, McDonnell M (2021). Three-dimensionally printed surgical simulation tool for brain mapping training and preoperative planning. Oper Neurosurg (Hagerstown).

[13] Martinez JL, Damon A, Domingo RA, Valero-Moreno F, Quiñones-Hinojosa A (2021). Retrosigmoid craniectomy and suprameatal drilling-3-dimensionally printed microneurosurgical simulation: 2-dimensional operative video. Oper Neurosurg (Hagerstown).

